# Comparison of Octopus Semi-Automated Kinetic Perimetry and Humphrey Peripheral Static Perimetry in Neuro-Ophthalmic Cases

**DOI:** 10.1155/2013/753202

**Published:** 2013-07-15

**Authors:** Fiona J. Rowe, Carmel Noonan, Melanie Manuel

**Affiliations:** ^1^Department of Health Services Research, University of Liverpool, Brownlow Hill, Liverpool L69 3GB, UK; ^2^Department of Ophthalmology, Walton Centre for Neurology and Neurosurgery, Liverpool L9 7LJ, UK; ^3^Department of Ophthalmology, Aintree Hospital University Trust, Liverpool L9 7AL, UK

## Abstract

*Aim*. To compare semikinetic perimetry (SKP) on Octopus 900 perimetry to a peripheral static programme with Humphrey automated perimetry. *Methods*. Prospective cross-section study comparing Humphrey full field (FF) 120 two zone programme to a screening protocol for SKP on Octopus perimetry. Results were independently graded for presence/absence of field defect plus type and location of defect. *Results*. 64 patients (113 eyes) underwent dual perimetry assessment. Mean duration of assessment for SKP was 4.54 minutes ±0.18 and 6.17 ± 0.12 for FF120 (*P* = 0.0001). 80% of results were correctly matched for normal or abnormal visual fields using the I4e target versus FF120, and 73.5% were correctly matched using the I2e target versus FF120. When comparing Octopus results with combined I4e and I2e isopters to the FF120 result, a match for normal or abnormal fields was recorded in 87%. *Conclusions*. Humphrey perimetry test duration was generally longer than Octopus SKP. In the absence of kinetic perimetry, peripheral static suprathreshold programme options such as FF120 may be useful for detection of visual field defects. However, statokinetic dissociation may occur. Octopus SKP utilising both I4e and I2e targets provides detailed information of both the defect depth and size and may provide a more representative view of the actual visual field defect.

## 1. Introduction

Visual field assessment is a valuable test in the neuro-ophthalmology clinic for determining presence of visual field deficit, aiding localisation of pathological lesion, and for recording improvement, stabilization, or deterioration of the underlying condition. Both kinetic and static perimetry options are frequently used in neuro-ophthalmology clinics. Static perimetry is often undertaken with the Humphrey automated perimeter (Humphrey Instruments, Dublin, CA), whilst kinetic perimetry has most commonly been undertaken using the Goldmann manual perimeter (Haag Streit, Switzerland). Both options, when directly compared, have been shown to reliably detect visual field loss [1–6]. Central static programmes such as the SITA 30-2 strategy have been used most with Humphrey perimetry in these studies. However, there is less information regarding the use of peripheral static programmes using Humphrey perimetry. Semiautomated kinetic perimetry (SKP) has been further developed in recent years, most notably with the Octopus 900 perimeter (Haag Streit, Switzerland). 

Assessment of the peripheral visual field in addition to assessment of the central field is often required in the evaluation of patients attending neuro-ophthalmology clinics. However, there is limited information available on the comparison of different peripheral visual field programmes. Thus, the purpose of this study was to directly compare SKP using the Octopus 900 perimeter to a peripheral static programme using the Humphrey automated perimeter in a neuro-ophthalmology clinic. 

## 2. Methods and Materials

A prospective cross-section study was undertaken with local ethical approval and in accordance with the Tenets of the Declaration of Helsinki.

### 2.1. Patients

Participants were not preselected for the study but were identified randomly; that is, notes were taken consecutively from the list waiting for visual field assessment without prior knowledge of patient ability and cognition. A selection bias existed in that the patients recruited to this study had been booked to an out-patient neuro-ophthalmology clinic for perimetry. Thus, there was an assumption that these patients had sufficient ability and cognition to undertake standard automated perimetry. 

Inclusion criteria were adult patients aged 18 years or older attending for visual field assessment, sufficient motor ability to sit at the perimeter unaided, able to press the response button, sufficient cognitive ability to understand and follow instructions for performing the test, willingness to undertake testing on both perimeters on the same day, and able to respond to both I4e and I2e target stimuli on Octopus perimetry. The exclusion criteria were patients with visual acuity less than 1.0 logMAR, those unable to sit for the duration of perimetry assessment, follow instructions for performing the test, or too ill to complete the full assessment. All patients underwent perimetry following full explanation of the purpose of the test and procedure. 

### 2.2. Visual Field Protocol

The full field 120 (FF120) two zone programme was used as the peripheral static programme on the Humphrey perimeter. This programme consists of 120 stimulus locations with a higher density of locations in the nasal than temporal visual field. The two zone strategy records locations as seen or unseen only. There is no determination of relative defect at abnormal points. 

**Figure 1:**
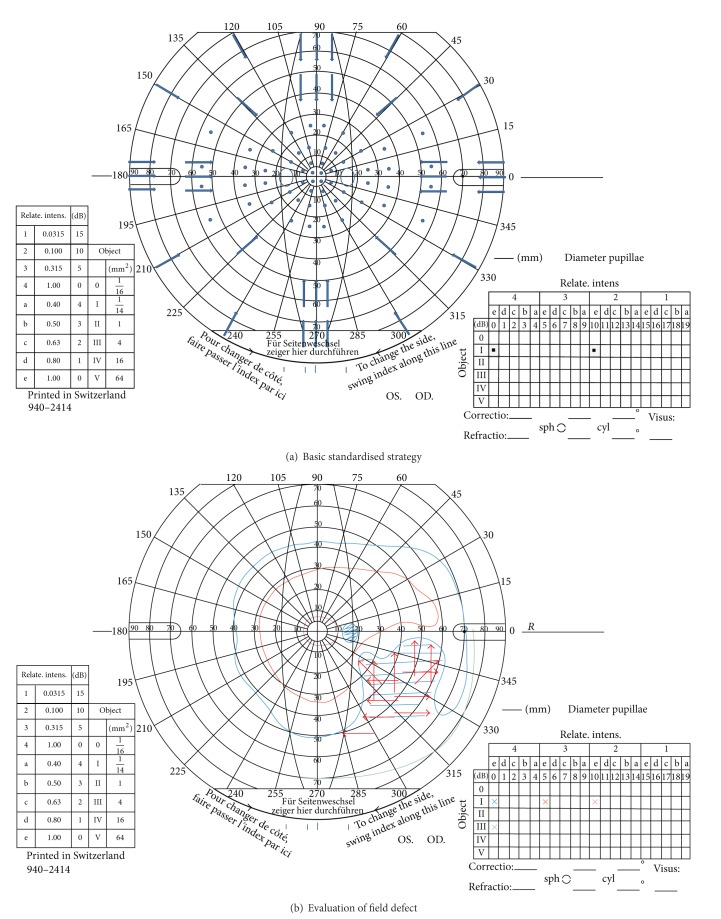
(a) The outer blue arrows depict the trajectory for I4e stimuli, and the inner blue arrows depict the trajectory for I2e stimuli. The spots indicate the position of static stimuli presentations. (b) An example of a visual field result with right-sided inferior partial quadrantanopia. The red arrows depict the trajectory for additional stimuli to plot the boundaries of the visual field defect.

For the purposes of standardisation and comparison in this study, a screening protocol was used for SKP. Two stimuli of the same size (0.25 mm^2^) were used but of different intensity (I4e, 1000 apostilbs and I2e, 100 apostilbs). The peripheral visual field boundary and blind spot were assessed using a size I4e target. Central visual field boundary was assessed using a size I2e target. A minimum of twelve vectors were assessed for the peripheral visual field and eight for the central visual field inclusive of vectors offset from the vertical and horizontal meridia moving centripetally, similar to previously reported testing strategies [7, 8]. Following assessment, the response points along each vector were joined to form the isopter for I4e and I2e targets, respectively. In addition, 56 static points (14 per quadrant) were assessed within the central 30 degrees of the visual field using the I4e target (Figure 1). Movement of the target on the Octopus perimeter was set at 5°/sec for determination of central and peripheral isopter boundaries and at 3°/sec for determination of the blind spot boundary and quantification of boundaries of visual field defects. 

The study protocol consisted of visual field assessment with both Humphrey and Octopus perimetry on the same day. The order of testing was randomised as to which of the two assessment types was used first in order to take fatigue effect into consideration. A short break of 5–10 minutes was allowed between testing on either perimeter. Randomisation was not undertaken using a computer generated table. Patients were assigned to one perimeter or another according to which perimeter was available for use at the time the patient was called for assessment. 

Reliability was determined automatically by fixation loss and false positive and false negative responses on Humphrey perimetry and by manually checking false positive and false negative responses on Octopus perimetry. Poor reliability was deemed present with fixation losses and false positive and false negative responses of >25% [9].

### 2.3. Comparison of Results and Statistical Analysis

Visual field results in both groups were assessed for presence or absence of visual field defects. Full (normal) visual fields by kinetic assessment were defined as visual field results with isopters for I4e and I2e falling within age-matched ranges and no focal defects within the isopter area (apart from the blind spot in the temporal field). Visual field loss was defined as isopter boundaries constricted within the age-matched ranges which could be global constriction or a defect type. Defect types were classified according to a modified list based on those reported by Pineles et al. [10] and the Ocular Hypertension Treatment Study (OHTS) [11] and outlined in Table 1. We added a category of functional visual field loss where the visual field defect followed a tubular or spiral pattern on testing.

One author assessed the results of Octopus perimetry (FR) and the second author assessed the results of Humphrey perimetry (CN). Each reviewer was masked to patient identifiers and to the classification by the other reviewer. Further independent assessment of a sample of visual field results (*N* = 36) was made by the third author (MM) who determined whether the paired Humphrey and Octopus results were a match or not.

A direct comparison was made for Octopus and Humphrey perimetry results using the statistical package SPSS version 19 (IBM SPSS Statistics, USA). Duration of test was compared between perimeters using unpaired *t* tests. Bland-Altman strategy was used to compare the differences between two independent measurements for duration of test versus the average test duration. When analysing the Bland-Altman results, we expected most of the differences to lie within ±1.96 SD if normally distributed. Provided the differences within ±1.96 SD would not be clinically important, we considered that the two methods can be used interchangeably. We therefore set a clinical cutoff of within 1 minute as a clinically acceptable difference between perimeter test durations.

The chi-square test (*χ* ^2^) was used to evaluate correlation between detection of normal and abnormal test results by either perimeter. Kappa (*κ*) evaluation of agreement was used to correct the proportion of agreement between perimeters due to chance when evaluating intraobserver interpretation of visual field results. *κ* values range from 0 to 1. A *κ* value of 1 was defined as perfect agreement, and a value of >0.7 was deemed a strong agreement [12].

## 3. Results

Sixty-nine patients attending neuro-ophthalmology clinics underwent dual testing with Humphrey FF120 and Octopus SKP perimetry during the same clinic visit. Five patients were subsequently excluded due to Octopus perimetry being undertaken using a size III4e target or I4e target only. 

Sixty-four remaining patients (113 eyes) had diagnoses of posterior visual pathway pathology, anterior visual pathway pathology, functional impairment of visual field, and “normal” findings (Table 2). Thirty-one patients had diagnoses that were classed as neurological defects (postchiasmal) and 28 patients that were classed as ocular defects (prechiasmal). Five patients had normal visual fields or nonspecific visual field defects classed as functional or spurious. There were 29 females and 35 males with a mean age of 48 years (SD 14). All patients were able to respond to both I4e and I2e target stimuli. On Humphrey perimetry the central and peripheral reference decibel level was a mean of 32.6 (SD 2.4).

### 3.1. Duration of Assessment

**Figure 2:**
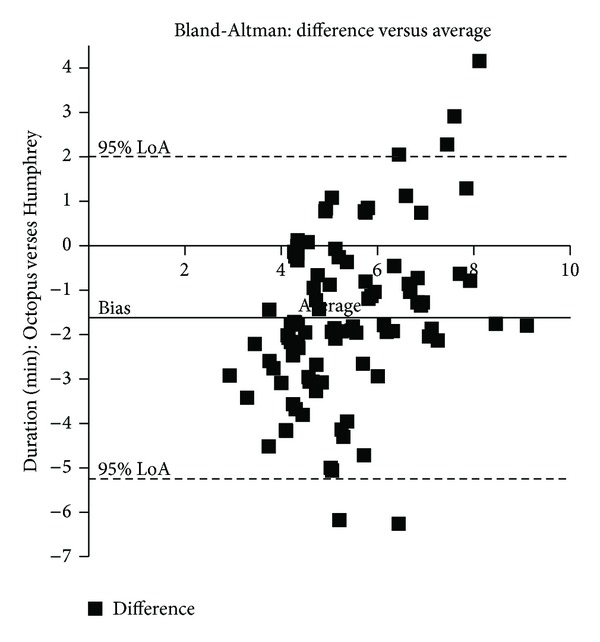
Duration of assessment. The solid line represents the mean bias of −1.62 with a higher mean test duration for Humphrey perimetry compared to Octopus perimetry. The dotted lines represent ±1.96 SD (−5.25 to 2.01). Variability increases with longer test duration averages with Octopus perimetry having longer test times than Humphrey perimetry and vice versa.

The mean duration of assessment for SKP was 4.54 minutes ±0.18 compared to the mean duration for Humphrey perimetry of 6.17 ± 0.12 which was significantly different (*P* = 0.0001 unpaired *t* test). Although the mean duration was higher for Humphrey perimetry (difference between means of −1.63 ± 0.22), Bland Altman analysis showed proportional change when the differences were compared between the two perimeters (Figure 2). The confidence intervals ranged from −5.25 to 2.01 minutes with differences exceeding our clinical cutoff of within 1 minute. With larger variances, SKP showed longer test durations than Humphrey perimetry (16 eyes (15%), Table 3).

### 3.2. Comparison of Octopus Perimetry to Humphrey Perimetry

80% of results (90 eyes) were correctly matched for normal or abnormal visual fields using the I4e target versus Humphrey FF120 (Table 4), and 73.5% (83 eyes), correctly matched using the I2e target versus Humphrey FF120 (Table 5). Mismatch was due to the I4e isopter being classed as normal or showing only a partial defect or different defect to Humphrey perimetry. Mismatch with the I2e target was due to the isopter being classed as normal, showing a different defect, partial defect, or being more constricted. In three eyes only, the Humphrey result was classed as normal while the Octopus result was classed as showing a visual field defect. In all other discrepancies, the Humphrey result was worse. 

When comparing the Octopus field with combined I4e and I2e isopters (i.e., either or both targets detecting a defect) to the Humphrey result, a match for normal or abnormal fields was recorded in 87% (98 eyes). Nine eyes (8%) had mismatching field defects from perimeter results. Three eyes (2.6%) had normal Humphrey results and abnormal Octopus results, while three eyes (2.6%) had normal Octopus results and abnormal Humphrey results. The features of these match discrepancies are outlined in Table 6.

On independent grading of a sample of results, 80% of results (28 eyes) were correctly matched for normal or abnormal visual fields using the I4e target versus Humphrey FF120 (Table 7), and 80% (28 eyes) were correctly matched using the I2e target versus Humphrey FF120 (Table 8). When comparing the Octopus field with combined I4e and I2e isopters to the Humphrey result, a match for normal or abnormal fields was recorded in 83% (29 eyes). The agreement between matching results by the first two authors versus independent matching by the third author was significant (*P* = 0.0001*χ* ^2^) with 30 of 35 results being correctly matched (*κ* = 0.8). Of the five results not correctly matched, each had been matched as abnormal for Humphrey and Octopus perimetry by the first authors but with a mismatch (Humphrey normal and Octopus abnormal in four results, Humphrey abnormal and Octopus normal in two results) by the third author.

For all comparisons, the Humphrey result was classed as showing a worse field (greater size of field defect) in 38 eyes. Conversely, the Octopus result was classed as showing a worse field in 20 eyes which was significantly less than Humphrey perimetry, *P* = 0.001 (*χ* ^2^ test). There was no significant difference for Humphrey or Octopus results being worse in abnormal visual field results due to either ocular or neurological causes (*P* = 0.77 and *P* = 0.964, respectively, *χ* ^2^ test).

## 4. Discussion

During Humphrey FF120 perimetry there is an initial determination of central and peripheral threshold levels (calculated in decibel values: dB) at the beginning of the test. This is used to determine the individual's reference hill of vision and stimuli are subsequently presented at the predetermined test locations at six decibel intensities higher than the expected threshold for each location. A mean reference level of 32.6 dB (SD 2.4) was calculated for the Humphrey results in this study. Thus, stimuli intensities would range from a mean of approximately 26 dB.

For Octopus SKP we used the I4e target to determine the peripheral boundary of the visual field and I2e for the central boundary. Calibration of the Octopus with a background luminance of 31.4 apostilbs and 1000 apostilb maximum stimulus luminance results in a dB value of 20 for the I4e target and 30 dB for the I2e target. 

Notably, the decibel scale is not standardised across the Humphrey and Octopus perimeters as the maximum luminance varies between the two perimeters. 

On comparison of results, a correct match of visual field result was 80% for the I4e target and 73.5% for the I2e target on SKP in comparison to the visual field result on Humphrey perimetry. Furthermore, when both I4e and I2e isopters were compared, in conjunction with each other, to the Humphrey result, a correct match was recorded for 87% of results. Thus, combined assessment of the peripheral and central field with Octopus perimetry led to the more sensitive detection of visual field deficit. A mismatch of results occurred for 15 eyes (13%). Eight percent of comparisons both showed abnormal visual field results but lacking an accurate match of defect with abnormalities mainly relating to constriction of field versus a defect in different quadrants of the visual field. Three eyes (2.6%) had normal Humphrey results but corresponding Octopus results showed peripheral superior defects (all in cases of pituitary adenoma). In a further three eyes (2.6%) normal Octopus results were recorded but Humphrey results showed abnormalities. It should be noted that in two results, the Humphrey result showed involvement of spurious points or generalised constriction but in which no specific diagnosis of visual field type could be made. Thus, these results could represent normal visual fields in which the patient failed to make adequate responses to stimuli from time to time. Given these comparisons of mismatch, we did not feel that the Octopus missed more defects than Humphrey, or vice versa. Independent grading of a sample of visual field results provided similar match comparisons, and a strong agreement for matched results by the first two authors in comparison to the third author was found. 

Previous comparative studies have contrasted SKP with static perimetry within the central 30 degrees in ocular diseases such as advanced glaucoma, optic neuritis, and optic nerve head drusen, with good comparisons and test-retest reliability. Furthermore, improved defection of visual field loss was obtained when both tests were used in conjunction with each other [1–4, 13]. Similar comparisons for neuro-ophthalmic cases have been reported with equal reliability in 77% of eyes [5], and our results are similar to these with SKP compared to Humphrey FF120 peripheral static perimetry. 

Disadvantages of static perimetry have been reported as inaccurate location of lesion to the anterior visual pathway, failure to detect macular sparing hemianopia, and overestimation of visual field extent [6]. We found the latter in our comparisons. When comparing all matched visual field results, the Humphrey results were graded as being more extensive than Octopus results in 38 eyes, and the Octopus results were graded as being more extensive than Humphrey results in 20 eyes. The difference of Humphrey results being more extensive than Octopus results was significant and might reflect the presence of statokinetic dissociation which has been defined as the static defect being larger than the kinetic defect [14, 15]. Statokinetic dissociation has been reported as occurring as a physiological phenomenon and has been found to increase towards the periphery of the visual field and decrease towards the centre of the visual field [15]. We did not find Statokinetic dissociation to be more prevalent in neurological versus ocular causes of visual field loss. 

A previous comparison of semi-kinetic perimetry versus automated central static threshold perimetry reported a median test duration of 13 minutes for the kinetic option and 11 minutes for the static option [2]. We found the opposite in our study with a mean test duration of 4.54 minutes for SKP and 6.17 minutes for Humphrey static perimetry. This may reflect the different number of isopters and vectors assessed for Octopus perimetry but also the use of a peripheral suprathreshold static programme rather than a central threshold static programme. 

On further evaluation of the individual test durations versus the average test duration, there was a wide variability, and the Humphrey test was not consistently longer than the Octopus test. Although we used a screening assessment for Octopus perimetry to standardise the initial outline of the visual field, we added more vectors to further define visual field defect boundaries (as described in the methods). The SKP screening assessment also incorporated static assessment of the visual field similar to previous studies using Goldmann perimetry with Armaly-Drance style strategies [7, 8]. Thus, the Octopus test became more detailed in the presence of more complex visual field defects. This may explain the crossover of test duration evident on Bland-Altman analysis in which the Octopus test duration was longer than the Humphrey test duration in 16 eyes (15%). The Humphrey FF120 programme utilised a two zone strategy in which stimuli were recorded as either seen or unseen and would not provide any detailed information with respect to the depth of visual field defect. In these cases, it could be argued that Octopus perimetry provides more detailed and informative evaluation of the visual field with better representation of the field defect in terms of its relative or absolute defect severity and which may be more representative of the individuals field defect that was shown by automated perimetry. Further evaluation of this aspect in conjunction with patient reported outcome measures for impact of visual field on activities of daily living and quality of life would be useful. 

There are some limitations to our study. Although the cases recruited to this study were representative of the types of pathology and visual field defects seen in our neuro-ophthalmology clinics, a larger sample of posterior versus anterior visual pathway defects would have allowed greater comparisons of differences between SKP versus static perimetry. Our comparison of 28 patients with ocular pathology to 31 patients with neurological pathology did not show any significant differences. 

A comparison of the FF120 peripheral strategy to a central threshold strategy would be useful to determine if the central threshold static programmes indicate the presence of visual field defects that may impinge more on peripheral than central visual field, such as in cases of pituitary adenoma. 

## 5. Conclusions

This study demonstrates that the combined Octopus I4e and I2e targets were more sensitive to detection of visual field loss than either target alone. Generally Humphrey perimetry test duration was longer than Octopus SKP although this was not consistent for all tests. When a more detailed evaluation with Octopus SKP was undertaken, this was at times longer than the Humphrey assessment. In the absence of kinetic perimetry options in neuro-ophthalmology clinics, peripheral static suprathreshold programme options such as the FF120 may be useful for detection of visual field defects. However, it must be noted that Statokinetic dissociation can occur with static perimetry. Octopus semi-kinetic perimetry utilising both the I4e and I2e targets provides detailed information of both the defect depth and size and may provide a more representative view of the actual visual field defect, particularly for more moderate to severe visual field defects.

## Figures and Tables

**Table 1:** Classification of visual field abnormalities.

Visual field classification	Number of results (total 113 eyes)
Normal	7
Altitudinal defect	
Arcuate defect	
Constriction (widespread)	23
Functional	3
Homonymous hemianopia	33
Bitemporal hemianopia	4
Inferior defect	6
Nasal step	4
Quadrantanopia (inferior)	4
Quadrantanopia (superior)	20
Scotoma (central)	2
Scotoma (paracentral)	
Superior defect	7
Temporal wedge	
Vertical step	

**Table 2:** Diagnosis of pathology.

Type of pathology	Type of visual field impairment
Posterior visual pathway (stroke, pituitary adenoma, arteriovenous malformation, and tumour metastases)	Homonymous hemianopia
	Homonymous quadrantanopia
	Bitemporal hemianopia
	Bitemporal quadrantanopia

Anterior visual pathway (papilloedema, optic neuritis, and idiopathic intracranial hypertension)	Enlarged blind spot
	Constriction

Functional—no pathology detected on investigation	Constriction
	Spurious loss—nonspecific

Normal—no pathology detected on investigation	No visual field loss

**Table 3:** Test duration: Octopus greater than Humphrey.

Visual field defect type	Number of eyes
Bitemporal hemianopia	3
Homonymous hemianopia	3
Partial quadrantanopia	3
Constricted visual field	2
Superior defect	2
Nasal loss	1
Enlarged blind spot	1
Functional, nonspecific	1

	Total: 16 eyes

**Table 4:** I4e outcome classification for Octopus and Humphrey results.

Count	Crosstab		
	Humphrey outcome		Total
	Normal	Abnormal	Normal
Octopus outcome I4e			
Normal	9	10	19
Abnormal	1	81	82
Mismatched defect	0	12	12

Total	10	103	113

Chi^2^, *P* = 0.0001.

Kappa = 0.35.

**Table 5:** I2e outcome classification for Octopus and Humphrey results.

Count	Crosstab		
	Humphrey outcome		Total
	Normal	Abnormal	Normal
Octopus outcome I2e			
Normal	8	16	24
Abnormal	2	75	77
Mismatched defect	0	12	12

Total	10	103	113

Chi^2^, *P* = 0.0001.

Kappa = 0.56.

**Table 6:** Mismatched perimetry result features.

Abnormal Humphrey and Octopus visual field results	Normal Humphrey and abnormal Octopus visual field results	Abnormal Humphrey and normal Octopus visual field results
Mismatched defects—defects in different quadrant *N* = 1	Peripheral superior defect on Octopus perimetry *N* = 3	Spurious missed points on Humphrey perimetry (nonspecific) *N* = 1
Constricted field versus spurious missed points *N* = 2		Peripheral nasal defect on Humphrey perimetry *N* = 1
Nasal defect versus spurious missed points *N* = 1		General constriction of field on Humphrey perimetry *N* = 1
Constricted field versus nasal defect *N* = 3		
Constricted field versus inferior defect *N* = 1		
Constricted field versus superior defect *N* = 1		

Total = 9 (8%)	Total = 3 (2.6%)	Total = 3 (2.6%)

**Table 7:** I4e outcome classification for Octopus and Humphrey results (assessor 3).

Count	Crosstab		
	Assessor 3 Humphrey outcome		Total
	Normal	Abnormal	Normal
Assessor 3 Octopus outcome I4e			
Normal	3	3	6
Abnormal	4	25	29

Total	7	28	35

Chi^2^, *P* = 0.044.

Kappa = 0.45.

**Table 8:** I2e outcome classification for Octopus and Humphrey results (assessor 3).

Count	Crosstab		
	Assessor 3 Humphrey outcome		Total
	Normal	Abnormal	Normal
Assessor Octopus outcome I2e			
Normal	3	3	6
Abnormal	4	25	29

Total	7	28	35

Chi^2^, *P* = 0.044.

Kappa = 0.21.
